# Genomic divergence reveals unique populations among Indian Yaks

**DOI:** 10.1038/s41598-020-59887-3

**Published:** 2020-02-27

**Authors:** Jayakumar Sivalingam, M. R. Vineeth, T. Surya, Karanveer Singh, S. P. Dixit, S. K. Niranjan, M. S. Tantia, I. D. Gupta, D. Ravikumar

**Affiliations:** 1 ICAR-National Bureau of Animal Genetic Resources, Karnal, Haryana India; 2 Animal Genetics & Breeding Division, ICAR-National Dairy Research Institute, Karnal, Haryana India

**Keywords:** Phylogenetics, Chromosomes

## Abstract

The present study focused upon identification of genome-wide SNPs through the reduced representation approach and to study the genomic divergence of the Indian yak populations. A total of 80 samples belonging to Arunachali yak (N = 20), Himachali yak (N = 20), Ladakhi yak (N = 20) and Sikkimi yak (N = 20) of India were used in the study. The results of the study revealed a total of 579575 high quality SNPs along with 50319 INDELs in the Indian yaks. The observed heterozygosity was found to be high in Himachali yak, followed by Arunachali yak, Ladakhi yak and Sikkimi yaks. The Sikkimi yaks was found to be genetically distant, followed by Ladakhi yaks which was observed to have some few individuals from Arunachali and Himachali yaks. Arunachali and Himachali yaks are found to get clustered together and are genetically similar. The study provides evidence about the genomic diversity in the Indian yak populations and information generated in the present study may help to formulate a suitable breeding plan for endangered Indian yaks. Moreover, the unique yak populations identified in the study will further help to focus attention for future characterization and prioritization of the animals for conservation purposes through the ddRAD approach.

## Introduction

Yak (*Peophagus grunniens*) is a unique bovine species living in the difficult terrains, which appeared some two million years ago and provides the indigenous people with meat, milk, butter, cheese, wool, fiber, leather, fuel and travel requirements. Yaks have got adapted to very high altitude regions where the oxygen content is very low and the genes responsible for the adaptation to hypoxic conditions has already been reported^1^. Indian yaks are classified into Arunachali yak, Himachali yak, Ladakhi yak and Sikkimi yak based upon the geographical area inhabitation and so far, only Arunachali yak has been described as a breed in India. The population of yaks in India as per the 19^th^ Livestock census is 0.077 million^2^. In case of yaks, which are reared under high altitudes, data recording is almost nil or not being followed. Moreover, only few bulls are being maintained by the Brokpas or farmers who raise yaks which lead to the high level of inbreeding. Use of genome-wide SNPs to study the genetic diversity will have a clear advantage over use of only few microsatellite markers. SNPs are nucleotide variations in the DNA sequence of individuals of a population and are the most abundant molecular marker in the genome. However, the introduction of single nucleotide polymorphisms arrays made the collection of genome-wide marker data feasible at a fairly affordable costs^3^. The SNPs used in the array may not be geographically representative that results in ascertainment bias and inherently excludes detection of rare or population-specific variants^3^, a major source of information for both population history and genotype-phenotype association. This may impair estimation of population parameters like diversity, population subdivision, recombination and the identification of causal mutations. The recent advances in DNA sequencing technologies have facilitated the development of more efficient and cost effective strategies that allow simultaneous discovery and genotyping of SNPs in multiple individuals. The cost for genotyping 48 samples using Illumina Bovine SNP HD DNA Analysis is Rs 1547680^4^ whereas for the double digestion restriction-associated DNA (ddRAD) Sequencing of 24 samples is Rs 360000^5^. The recent ddRAD method have advantages over other SNP genotyping platforms as it reduces the ascertainment bias and can be developed easily for genotyping the SNPs of predetermined area over many samples in cost effective manner. The SNPs genotyped by this method could be used for studying genetic diversity, genetic introgression^6^, QTL mapping, genomic selection^7,8^, genome-wide association studies (GWAS)^9^, population studies and identification of selective sweeps^10^. Hence, in the present study, genome-wide SNPs identified through the reduced representation approach (ddRAD) were used to study genomic diversity as well the genetic polymorphism in the candidate genes responsible for high altitude adaptation in the Indian yak populations.

## Results

### Quality control processing

The restriction enzymes Sph I and MluC I were used for preparation of genomic library which resulted in the fragment size of 100 bp. A total of 259.674820 million raw reads were generated by the illumina Hiseq 2000. After processing the raw reads for it’s quality, a total of 242.801028 million good quality reads were obtained which accounted for 93.5% of total raw reads.

###  Alignment with reference genome

The QC processed good quality reads were aligned with Yak genome (*Bos mutus;* assembly BosGru_v2.0). A total of 95.4% of QC processed reads were mapped to the reference genome. Of the total aligned reads 49.17% were uniquely aligned to the reference genome. The QC processed quality reads covered 9.43% of the reference genome.

### SNP identification and annotation

The variants identified using SAMtools were quality filtered at read depth of 2, 5 and 10 along with phred-like consensus quality score of ≥30. The number of SNPs and INDELs identified at RD2 (Read Depth2), RD5 (Read Depth5)and RD10 (Read Depth10) in the different Indian yak populations are presented in Table 1. Similarly, the number of non-overlapping SNPs and INDELs identified at RD5 and RD10 in the Indian yak populations are presented in Table 2. Non-overlapping SNPs genotyped across all the samples at RD10 present in the Indian yak populations were filtered (Table 3) and were further used for studying the genetic distance and genomic diversity estimation.

**Table 1:** Number of SNPs and INDELs identified in different Indian yak populations.

Population	Variants					
	SNPs			INDELs		
	RD2	RD5	RD10	RD2	RD5	RD10
Arunachali	294593	269342	256051	25014	23194	21892
Himachali	283880	260572	241934	23582	21752	19935
Ladakhi	390342	352140	312518	27773	25176	21954
Sikkimi	201014	178435	150425	15596	14035	11710

**Table 2:** Number of SNPs and INDELs identified in Indian yak at various read depths.

Variants	Read depth		
	RD2	RD5	RD10
SNPs	952942	638980	579575
INDELs	77391	56245	50319
Total	1030333	695225	629894

**Table 3:** Number of non-overlapping SNPs genotyped across all the samples at RD10 in Indian yak.

Variants	Count
SNPs	25177
INDELs	1291
Total	26468

The high quality SNPs identified against yak reference genome at read depth ≥10 and quality ≥30 were annotated using the SnpEff software. Based on the sequence ontology terms, 425292 of SNPs were present in the intronic region; 429945 of SNPs in transcript region; 661221 of SNPs in intergenic region and 6532 of SNPs in exonic region (Table 4).

**Table 4:** Annotation of SNPs in Indian yak.

Type	Count
Downstream	93803
Exon	6532
Intergenic	661221
Intron	425292
Splice_Site_Acceptor	26
Splice_Site_Donor	40
Splice_Site_Region	571
Transcript	429945
Upstream	95790
UTR_3_Prime	3255
UTR_5_Prime	656

The SNPs identified in the present study were found to have varying impacts and 215 SNPs were found to have high impact, 3388 SNPs have low impact, 2804 SNPs have moderate impact, and 1710724 SNPs have impact as modifier. The SNPs were also found to have number of effects by functional class and were found to be missense (2829 SNPs), nonsense (124 SNPs) and silent (2808 SNPs). The single base pair change in the DNA that differs from the usual base at a position has been studied (Table 5). In the present study due to DNA substitution a total of 7881879 transitions (t_s_) and 3329790 transversions (t_v_) were identified. The t_s_/t_v_ ratio in the present study was found to be 2.36.

**Table 5:** Base changes identified in Indian yak.

	A	C	G	T
A	0	39,654	154,575	36,150
C	46,312	0	36,891	165,677
G	165,475	36,658	0	46,645
T	35,353	155,084	39,566	0

### Annotation of the SNPs in the candidate genes for high altitude adaptation

The candidate genes for high altitude adaptation were selected from studies in cattle and yak^11,12^ and SNPs were annotated in the genes (Supplementary Material 1).

### Genetic diversity

**Figure 1:**
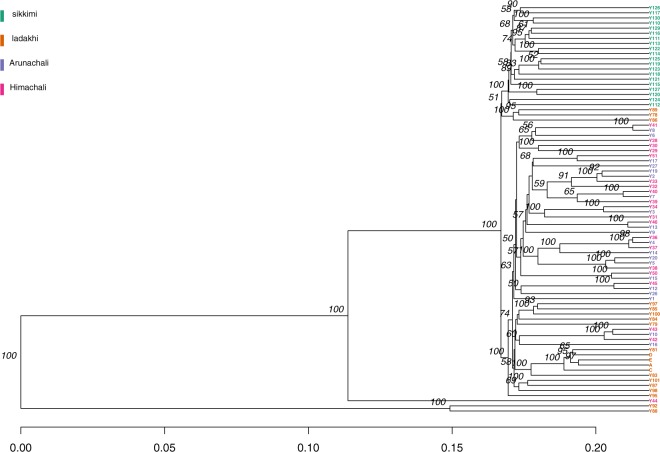
Phylogeny based upon genetic distance among the Indian yaks.

The observed and expected heterozygosity estimated in the Indian yak population ranged from 0.28 to 0.34 and 0.28 to 0.30 respectively (Table 6). The inbreeding coefficient (F_IS_) ranged from 0.01 to 0.02 (Table 6). The genetic distance estimated between Indian yak populations ranged from 0.0064 (Arunachali and Himachali) to 0.36812 (Sikkimi and Ladakhi) (Table 7) (Fig. 1). ddRAD results were found to be comparable with the microsatellite data for estimation of heterozygosity and inbreeding coefficient (Table 8).

**Table 6:** Genetic diversity in Indian yak.

Population ID	Private all eles	Number of samples	Obs. Hom	Std. Err	Exp. Hom	Std. Err	Obs. Het	Std. Err	Exp. Het	Std. Err	Pi	Std. Err	Fis	Std. Err
SIKKIMI	33	20	0.7169	0.0021	0.7183	0.0019	0.2831	0.0021	0.2817	0.0019	0.2889	0.0019	0.0159	0
LADAKHI	5	20	0.6664	0.0021	0.6927	0.0017	0.3136	0.0021	0.3073	0.0017	0.3152	0.0017	0.0226	0
ARUNACHALI	0	20	0.6638	0.0024	0.7019	0.0018	0.3362	0.0024	0.2981	0.0018	0.3058	0.0018	−0.0688	0
HIMACHALI	0	20	0.6565	0.0023	0.7006	0.0017	0.3435	0.0023	0.2992	0.0017	0.3068	0.0018	−0.0919	0

**Table 7:** Genetic distance among Indian Yak.

	Sikkimi	Ladakhi	Arunachali	Himachali
Sikkimi		0.368126	0.0437961	0.041001
Ladakhi			0.0276542	0.0250536
Arunachali				0.00640055

**Table 8:** Comparison of heterozygosity and inbreeding coefficient level based upon Microsatellite vs ddRAD data.

	Microsatellite^13^	ddRAD
Observed heterozygosity	0.369 to 0.819	0.2831 to 0.3435
Expected heterozygosity	0.413 to 0.732	0.2817 to 0.3073
F_IS_	−0.021 to 0.207	−0.0919 to 0.0226

### Genetic differentiation

AMOVA analysis revealed the SNP variations among yak populations (3.47%); within breed (97.48%) as well among populations within groups is −0.95% (Table 9).

**Table 9:** Analysis of molecular variance in Indian yak.

Source of variation	Degrees of freedom	Sum of squares	Variance components	Percentage of variation
Among groups	3	10673.062	52.65296	3.47
Among populations within groups	76	110319.250	−14.42796	−0.95
Within populations	80	118434.000	1480.42500	97.48
Total	159	239426.312	1518.65000	

## Discussion

Non-availability of recommended SSRs by FAO as well SNP chip for yaks make a constraint to study the genetic diversity as well to proceed for estimating the genomic breeding value and genomic selection. Previously, in order to assess the diversity of Indian yak population, FAO recommended cattle SSRs were used. In the study^13^, only 27 SSRs (90%) out of 30 SSRs amplified the yak genomic DNA and only 23 SSRs were found to be polymorphic in the Indian yak populations. In the present study, ddRAD approach for studying the genetic diversity in yak has been attempted for the first time and has been found to be a cost effective and efficient method to assess the genetic diversity in the livestock species.

Based upon the estimated genetic distance in the Indian yaks, Sikkimi yaks was found to be genetically distant, followed by Ladakhi yaks. Arunachali and Himachali yaks were found to get clustered together and observed to be genetically similar. Private alleles are present maximum in Sikkimi yak followed by Ladakhi yak which makes the Sikkimi yak population unique among the other populations. The genetic distance between the different yak populations was found to be in decreasing order in Sikkimi-Arunachali; Sikkimi-Himachali; Sikkimi-Ladakhi; Ladakhi-Arunachli; Ladakhi-Himachali and Arunachali-Himachali yak populations.

The low level of genomic divergence between the yak populations indicates it’s origin from the same ancestors. Moreover, the low genetic differentiation among the groups may be because of the gene flow due to the movement of animals across different geographical locations. The existing genomic divergence may be due to the adaptation of the animals over years and also may be due to the geographical isolation of the animals. Higher molecular variation (97.48%) within the Indian yak populations may partly be due to lack of selection pressure applied and large effective population size that can be corroborated with the higher genetic diversity within the population. In the present study, genome-wide SNPs identified in the Indian yaks has been structurally and functionally annotated (Supplementary dataset 1). The candidate genes for high altitude adaptation were reported to be responsible for adaptation to hypoxia, anaerobic metabolism, iron homeostasis and pulmonary hypertension in various species. The EPAS1/HIF-2α which is a transcription factor that belong to hypoxia-inducible factor signaling pathway was found to be involved high altitude adaptation in through its role in response to hypoxia, erythropoiesis, iron homeostasis, pulmonary hypertension^1,11,14–16^. ITPR1 gene is target of HIF-2α and is involved in high altitude adaptation mechanisms^12^. HIF-1α regulates the oxygen homeostasis in cells and also regulates glycolysis^17,18^. NOS2 gene is expressed during the high altitude hypoxia and may aid in improved pulmonary capacity in high altitude^19^. GLUT1 like glucose transporters are responsible for anaerobic metabolism^12^. The SNPs identified in the candidate genes for high altitude adaptation need to be explored further in large population for its role in the adaptation process.

The range of estimated heterozygosity (both observed and expected) were found to be less in ddRAD when compared to microsatellites, which gives a wider range. In ddRAD, use of methylation sensitive restriction enzymes removes the repetitive sequences which may result in lower estimate of genomic diversity. Moreover the SNPs identified in the coding regions as well in the UTR regions may also result in lower estimate of genomic diversity. In case of microsatellites the higher estimate may be because of it’s location in the intronic regions where lot of repetitive sequences are present and have higher number of variations than the coding regions. Higher amount of variations in the intronic regions usually results in more variations in the allele size in the populations which usually gives a higher estimate of genetic diversity. Being a reduced representation approach of the whole genome in which the repetitive sequences are removed, the ddRAD approach has a genome coverage of 9.43% in the present study which is supported by the genome coverage of 13.4–14.8% in buffaloes^20^, 4.66% in chickens^21^ and 13% in ducks^22^. Through the ddRAD approach, the unique yak populations identified in the study will help for future characterization and prioritization of animals for conservation purposes.

## Methods

### Sample collection and ddRAD sequencing

The animals for blood collection were randomly selected from the breeding tract of the different Indian yak populations. The blood sampling of animals were performed in accordance with the relevant guidelines and regulations as approved by Institutional Animal Ethics Committee (IAEC) of National Bureau of Animal Genetics Resources (NBAGR), Karnal. A total of 80 blood samples were collected (20 samples from each of the 4 yak populations, viz., Arunachali yak, Himachali yak, Ladakhi yak and Sikkimi yak of India). Genomic DNA from the blood samples were isolated using Phenol-Chloroform method^23^ and the quality of the genomic DNA was checked by agarose gel electrophoresis and quantity by the digital nanophotometer. Optical density (OD) was also determined as the ratio of OD260 and OD280 in each sample. The samples meeting the required QC parameters of more than 100 ng/ul concentration and having a OD260 by OD280 ratio of 1.7–1.9 was considered for library preparation using the standard protocols described for ddRAD^24^. The double digestion of genomic DNA (1 microgram) was carried out using Sphl and Mluc1 restriction enzymes and cleanup of the digested product was carried out using Ampure beads. Ligation of P1 (Barcoded) and P2 adaptors were done using T4 DNA ligase. Size selection of the product was done after 2% agarose gel electrophoresis. The PCR amplification was carried out to enrich and add the Illumina specific adapters and flow cell annealing sequences. Then final pooling and sequencing was carried out on the illumina Hiseq. 2000 and 100 bp paired end reads were generated.

### Identification and annotation of SNPs

The raw reads were demultiplexed using custom perl scripts to obtain sample specific reads. Up to one mismatch was allowed to demultiplex the sample data. The low quality bases and regions showing base bias at the start or end and was removed from the reads using Prinseq lite version 0.20.4^25^. The Illumina 5′ and 3′ adapter sequences were also removed. The reads were processed in STACKS^26^ for finding the RAD loci and to retain the reads with minimum phred score of 15. The index of the reference genome were build using Bowtie2–2.3.3.1^27^. The QC processed reads were aligned with yak genome^28^ (*Bos mutus*; assembly Bos Gru_v2.0) using Bowtie 2–2.3.3.1. During the alignment, the parameters were set to very sensitive local paired end alignment and the maximum fragment length including gaps as 1000 bp. Samtools/bcftools version 1.6^29^ were used for further processing of the reads and to call the variants. The variants identified were filtered for minimum read depth (at 2, 5 and 10); maximum read depth (D ≤ 1000) and base quality score (Q ≥ 30) using vcfutils varFilter of Samtools. In order to identify the extent of genome coverage by the reads obtained by reduced representation approach, SAMtool was used. High quality SNPs identified in the present study were annotated both structurally and functionally with the help of SnpEff ^30^ and SnpSift^31^. In SnpEff, the effect of SNPs were identified once the data has been downloaded and database has been built. The program loads the binary database and builds a data structure called “interval forest” to perform an efficient interval search. The effect of SNPs were grouped according to the region as exonic, intronic, upstream and downstream regions, splicing sites and intergenic regions. SNPs in the coding region were further grouped into missense, nonsense and silent SNPs. For gene wise annotation, the candidate genes for high altitude adaptation^11,12,32,33^ were collected. The genomic location of the candidate genes were drawn from the NCBI database and the SNPs present in that region were captured from the annotated VCF file using VCFtools^34^.

### Genetic distance and diversity

The SNPs observed to be in common for all the four populations were used for estimating the genetic distance (proportion of loci that were different) and genomic diversity using poppr package^35^. The observed and expected heterozygosities as well the level of inbreeding coefficient were estimated. Analysis of Molecular Variance (AMOVA) was also calculated using Arlequin software^36^ (ver 3.5.2.2).

### Disclaimer

Authors mentioned the name of the service providers solely for providing accurate information and does not imply any recommendation.

## Supplementary information

Supplementary Dataset 1.

## Data Availability

Data supporting this paper was generated by ICAR-NBAGR. The datasets generated in the current study has been deposited in NCBI (BioProject PRJNA577203).
